# Evaluating Overton and Altmetric as tools for tracking healthcare research use and impact on policy and practice: a descriptive study

**DOI:** 10.3310/nihropenres.13999.1

**Published:** 2025-07-16

**Authors:** Ruth Tunn, Fiona Alderdice, Marian Knight

**Affiliations:** 1 NIHR Policy Research Unit in Maternal and Neonatal Health and Care, National Perinatal Epidemiology Unit, University of Oxford Nuffield Department of Population Health, Oxford, OX3 7LF, UK

**Keywords:** altmetrics, bibliometrics, clinical guidelines, evidence, impact, Overton, policy research, research evaluation

## Abstract

**Background:**

Since 2010, the UK’s National Institute for Health and Care Research has funded a policy research unit (PRU) focused on maternal and neonatal health, with a remit to build an evidence base for policy and clinical practice in this field. We explored the usefulness of the platforms Overton and Altmetric as tools to gain insight into the use of PRU research evidence in policy and practice.

**Methods:**

We searched Overton and Altmetric using article DOIs to identify citations of PRU-funded articles in policy documents and clinical guidelines. We excluded citations of the research in lists of excluded evidence, academic journal articles, and unverifiable citations. To obtain a count of unique citing documents for each article, we merged multiple editions/versions, translations, and duplicates of the same document. We calculated latency from article publication date to citation date, and citation distribution over time. We also developed descriptive case studies to explore how the citing policy documents used highly-cited research evidence.

**Results:**

The 110 published articles reporting research funded by the PRU received 134 unique policy document and clinical guideline citations; 43/110 articles (39%) were cited in at least one document. Most citing documents were authored by organisations based in the UK (52/134) and other high-income countries. Intergovernmental organisations accounted for around 15% of citations (20/134). The median time from article publication to citation was 183 weeks (range 0.4–575 weeks). Citation contexts varied; use of the evidence in citing documents included provision of general background information, detailed summaries of findings, and support/rationale for specific clinical recommendations.

**Conclusions:**

Overton and Altmetric are useful tools for identifying and exploring the use of research evidence in healthcare policy and clinical guidance. However, citation analysis alone cannot provide the complete picture. The delay between evidence publication and use in policy warrants further investigation.

## Plain language summary

Billions of pounds are spent every year in the UK on healthcare research. It is important to know whether this research makes a difference to people’s lives, but it can be surprisingly difficult to find this out. For research to benefit patients and the public, the findings have to be used. This could involve the evidence being used to support government policies or the clinical guidelines that healthcare workers use when deciding how to treat patients. However, typically nobody tells researchers when their work is used like this.

To try to understand how our research group’s findings have been used, we tried out two online tools. These tools scan the internet to find any mentions of individual research articles in documents that are posted online. They look particularly at clinical guidelines and at policy documents, which include reports written by government departments and non-governmental organisations; working papers; evidence submissions to parliamentary committees; and meeting minutes.

We found that about 40% of research articles published by our group were mentioned in at least one policy document or guideline. There was a delay of almost four years on average between an article being published and it being mentioned in a document or guideline. When we looked at how the research was used in the policy documents and guidelines, we found this varied a lot: for example, sometimes it was used to provide general background, while in other cases it was used to support government policies and clinical recommendations that could affect patients directly.

This study helped us to understand how our research is used and how it might make a difference to patients and the public. However, we need to work to speed up the pathway from research to policy so that people can benefit from research findings as soon as possible.

## Background

An ultimate aim of healthcare research is to create benefit for patients, through improved treatments and healthcare systems. However, much of academic research assessment has focused on publications in academic journals as the main output of research, and citations in academic journals as a proxy for the impact of research ^1– 3^. Academic articles are easy to quantify, and established indexes allow for tracking of citations of publications within other journal articles ^2^. However, such metrics can be manipulated and can incentivise poor research practices ^4^, and citations in journal articles reflect the relevance of the research to science, but not necessarily to society. There is increasing recognition of a detrimental gap between research and policy, which limits the real-world impact of healthcare research ^5, 6^.

The UK’s research assessment exercise, the Research Excellence Framework (REF), has attempted since 2014 to evaluate the impact of research, which it defines as ‘an effect on, change or benefit to the economy, society, culture, public policy or services, health, the environment or quality of life, beyond academia’ ^7^. Changes to the REF for its next iteration in 2029 further emphasise the importance of impact, aiming to ‘foster the connection between research and societal impact, seeking to encourage high quality research with tangible benefits for society and the economy’ ^8^. The results of the REF inform the distribution of public funding to UK universities ^9^. Thus, to obtain and retain funding, both via the REF and from other funders, researchers are increasingly expected to demonstrate real-world impact of their work ^10, 11^.

In recent years, platforms have emerged that track citations, also referred to as attention or mentions, in sources other than traditional academic journals ^3^. Overton is a database of over 13 million policy documents from over 43,000 organisations. Founded in 2019, it offers the facility to search for policy documents that cite specific research articles ^12^. Altmetric identifies and quantifies online mentions of research outputs on platforms such as news outlets and social media, and also increasingly indexes citations in policy sources ^13^. As of November 2024, Altmetric also indexes citations of research articles in clinical guidelines as a separate category of citations ^14^. These platforms thus have potential to facilitate identification of research use that is more directly relevant to patients, healthcare systems and society ^15^.

To ensure the UK government and its arm’s-length bodies have access to high-quality evidence to inform health policymaking, the National Institute for Health and Care Research (NIHR) introduced its Policy Research Programme ^16^. This programme includes long-term funding for Policy Research Units (PRUs) in priority healthcare areas. Since 2010, the NIHR has funded a PRU focused on maternal and neonatal health, a collaboration led from the National Perinatal Epidemiology Unit at the University of Oxford with a remit ‘to conduct research and review existing evidence to improve the care given to women, their babies and their families by contributing to the evidence base for clinical practice and health-policy at the national and local level’ ^17^. PRU research comprises a mix of projects prioritised by an Oversight Group made up of representatives from the Department of Health and Social Care, arms-length bodies and senior researchers in anticipation of future evidence needs; and agreed responsive projects that provide evidence for policymakers as and when the need arises. While individual examples of real-world impacts of PRU projects have been documented ^18^, systematic evaluation of how evidence generated by the PRU is used in policy and clinical practice is lacking. Identifying real-world use of evidence has been highlighted as a need within policy research in particular, as research in this area is intended by design to rapidly and directly inform policy.

In this study, we aimed to explore the usefulness of Overton and Altmetric as tools to gain insight into the use of PRU research evidence in policy. To this end, we used these two platforms to identify and quantify citations in policy documents and clinical guidelines of research articles funded through the PRU for maternal and neonatal health. We also developed case studies to explore how the citing policy documents used research evidence, dissemination strategies used for highly cited articles, and attention in the non-academic media.

## Methods

### Patient and public involvement

Patients and the public were not involved in the design, conduct or choice of outcome measures of this study. Feedback on the findings was provided by the PRU-MNHC Co-Investigators Group, which includes patient and public involvement partners, prior to reporting; this informed the writing of the Discussion section of this article.

### Identification of outputs funded by the Policy Research Unit in Maternal and Neonatal Health and Care

The Policy Research Unit in Maternal Health and Care (PRU-MHC) was established in 2010 within the National Perinatal Epidemiology Unit at the University of Oxford. A second programme of work was funded in 2019 and saw the Unit renamed the NIHR Policy Research Unit in Maternal and Neonatal Health and Care (PRU-MNHC). The third, current, five-year programme started in 2024. Funding is provided by the Policy Research Programme at the Department of Health and Social Care (England) via the NIHR. For the purposes of this study, we focused on articles published in academic journals that reported research funded by the first or second PRU programme. We use ‘PRU’ throughout the paper to refer to the first and second programmes collectively, when not otherwise specified. When specifying a specific programme, we refer to the first programme as the PRU-MHC 2010–2018 programme and the second as the PRU-MNHC 2019–2023 programme.

The PRU holds a list of publications arising from projects completely or partially funded by each of the PRU programmes. This is maintained via mandatory internal reporting of articles by all PRU researchers at the point of publication. We used this list as the primary source of articles reporting PRU-programme-funded research. We also searched Scopus by funding number for articles that reference the grant code for either of the completed PRU programmes (108/0001 for the PRU-MHC 2010–2018 programme and PR-PRU-1217-21202 for the PRU-MNHC 2019–2023 programme). This search was conducted on 2 September 2024. Scopus is a proprietary research instrument and was used under institutional licence for this study. We cross-referenced the output of the Scopus search with the internally-held publication list to generate a definitive list and to identify any gaps in reporting of article publications.

### Search for citing policy documents and clinical guidelines

Overton and Altmetric are proprietary research instruments and were used under institutional licences for this study. Overton uses a broad definition of policy documents: ‘documents written primarily for or by policymakers that are published by a policy focused source’ ^19^. Clinical guidance is included as a document type, alongside the broad categorisation ‘publications’, within this definition. To identify policy documents that cite PRU-funded articles, we searched Overton by the digital object identifier (DOI) of each article. To identify any instances where DOI indexing failed, we also searched Overton by the title of each PRU-funded article, and cross-checked the output with that of the relevant DOI search. These searches were conducted on 17 September 2024.

Altmetric tracks mentions of published research in policy documents and clinical guidelines as separate categories. Policy documents are defined as ‘policy sources that are designed to change or otherwise influence guidelines, policy or practice’ ^20^. Clinical guidelines are defined as ‘documents that offer recommendations for clinicians and healthcare practitioners on how to manage and treat specific medical conditions or support decision-making in patient care’ ^21^. We searched Altmetric using the DOIs of all articles funded by each completed PRU programme, and filtered the results by source to return only ‘policy documents’ and ‘clinical guidelines’ based on Altmetric’s classifications. These searches were conducted on 2 December 2024.

### Full-text screening

One author (RT) reviewed every full-text document returned by the Overton and Altmetric searches to check the relevance of the document and the context in which the PRU-funded article was cited. Where documents were no longer available via the URL provided by Overton or Altmetric, we searched the Internet Archive’s Wayback Machine ( https://web.archive.org/) using the defunct URL to locate an archived version where possible.

We located citations of the PRU-funded article in each policy document by searching for the first author’s name and/or key words from the title to locate the article in the reference list, and searching for in-text citations based on the citation style of the document. We extracted the text where the article was cited to provide the citation context. We excluded the following:

- Policy documents that cited the PRU-funded article in a list of excluded sources
- Research or opinion articles published in academic journals (unless a clinical guideline or reporting development of a clinical guideline)
- Cochrane reviews
- National maternal and perinatal mortality surveillance reports (MBRRACE-UK reports)
- Policy documents in which no reference to the PRU-funded article could be located
- Policy documents that could not be located (either via the original URL or the Wayback Machine) to allow verification of the citation
- Apparent indexing errors by Overton (when searching for an article DOI returned a policy document, but the PRU-funded article was not cited and extracts supplied by Overton related to a different article)

We also excluded documents returned by Altmetric that were duplicates of those already identified by Overton.

During the full text screen, we also identified overlapping policy documents, defined as the following:

- Translations of documents
- The same document published by the same organisation in a different location
- The same document co-produced by multiple organisations and published separately by each organisation
- Part of a document (e.g., a single chapter) published separately and as part of a complete document
- Multiple editions or versions of the same document

**Figure 1.**
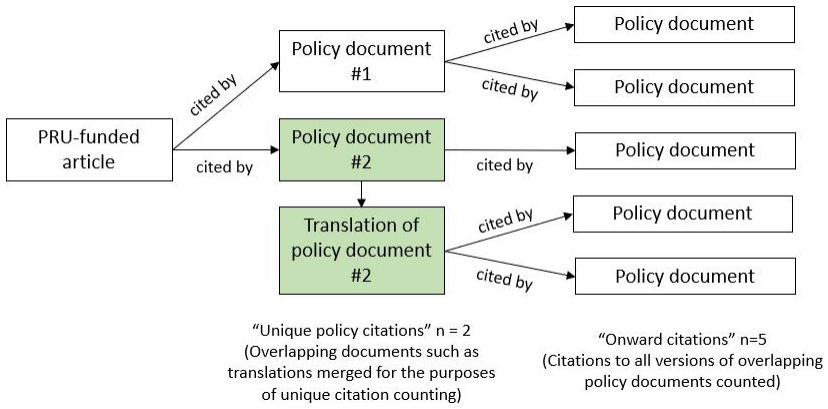
Terminology and citation counting: an example scenario.

We flagged these documents and excluded all but the earliest citation in each overlapping group from the count of unique policy documents citing PRU-funded articles, and from the analysis of citation date and latency. However, we extracted data from all versions of the documents and for results of the Overton search, we included all versions separately in analysis of onward citations (that is, later policy documents that cited the policy documents that cited PRU-funded articles, see Figure 1). Onward citation counts were not obtainable for results returned by Altmetric.

### Policy citation data extraction

For each included policy document, we extracted the following information from the search results output provided by Overton or Altmetric:

- bibliographic details of the article cited (title, publication date, DOI);
- bibliographic details of the policy document (title, date, URL);
- the name and country of the citing organisation;
- the type (government, intergovernmental organisation, think tank, other) of the citing organisation. However, as Altmetric does not categorise citations by the type of citing organisation, we added this information for Altmetric results based on review of the organisations’ websites;
- the type of document (policy publication, clinical guideline, working paper, blog post, other). ‘Policy publications’ included documents indexed by Overton as the document type ‘publications’ and those indexed by Altmetric as ‘policy document’; ‘clinical guidelines’ included documents indexed by Overton as ‘clinical guidance’ and by Altmetric as ‘clinical guidelines’.

For documents returned by Overton, we also extracted the number of onward citations of the document in later policy documents produced by the same and other organisations (this information is not provided directly by Altmetric as a search output).

We also obtained directly from the publisher’s website the dates that PRU-funded articles were first made available online, because in some cases this preceded the ‘publication date’ in a journal issue; and details of copyright licencing (non-open access, or Creative Commons license type).

### Quantifying citations in academic journal articles

We searched Scopus by the DOI of each PRU-funded article. We extracted the number of ‘citations in Scopus’ reported in the article metrics. These searches were conducted on 7 November 2024.

### Statistical analyses

All analyses were conducted in MS Excel. The first PRU funding round ended in 2018; the second ended in December 2023. Because of the time taken for articles to reach publication and the expected lag between article publication and citation in policy documents, we analysed the information for the two PRU funding rounds separately, as well as summarising the overall data for the two programmes to summarise the policy impact of the PRU as a whole.

We report the total number of unique citations of PRU-funded articles in policy documents, and summarise the number of unique citations per article as median and range. Citations are counted on a per-document basis i.e., the same article cited multiple times in the same document counts as a single “unique citation”. Two different articles cited in the same document count as two separate “unique citations”. We also report the total number of unique citations by country and type of citing organisation, and type of citing document. A geographic citation distribution map was created using Datawrapper ( https://app.datawrapper.de/).

We calculated the per-year and cumulative number of articles published and citations in policy documents over time and the latency from first availability of articles online to policy citation. Where documents overlapped in content and were merged for the count of unique citations, we used the earliest policy citation (as reported by Overton) as the citation date and to calculate latency. For documents identified by searching Overton, we also report the total number of onward citations in later policy documents ( Figure 1).

### Case studies

For the three individual PRU-funded articles with the highest number of citations in policy documents, we produced descriptive case studies. These include the number, countries, and types (organisation type/document type) of policy citations and examples of citation context. We also used Altmetric to identify the number of social media citations to the individual article and coverage in major news media outlets, and we describe any dissemination activities that were undertaken to publicise the article.

## Results

**Figure 2.**
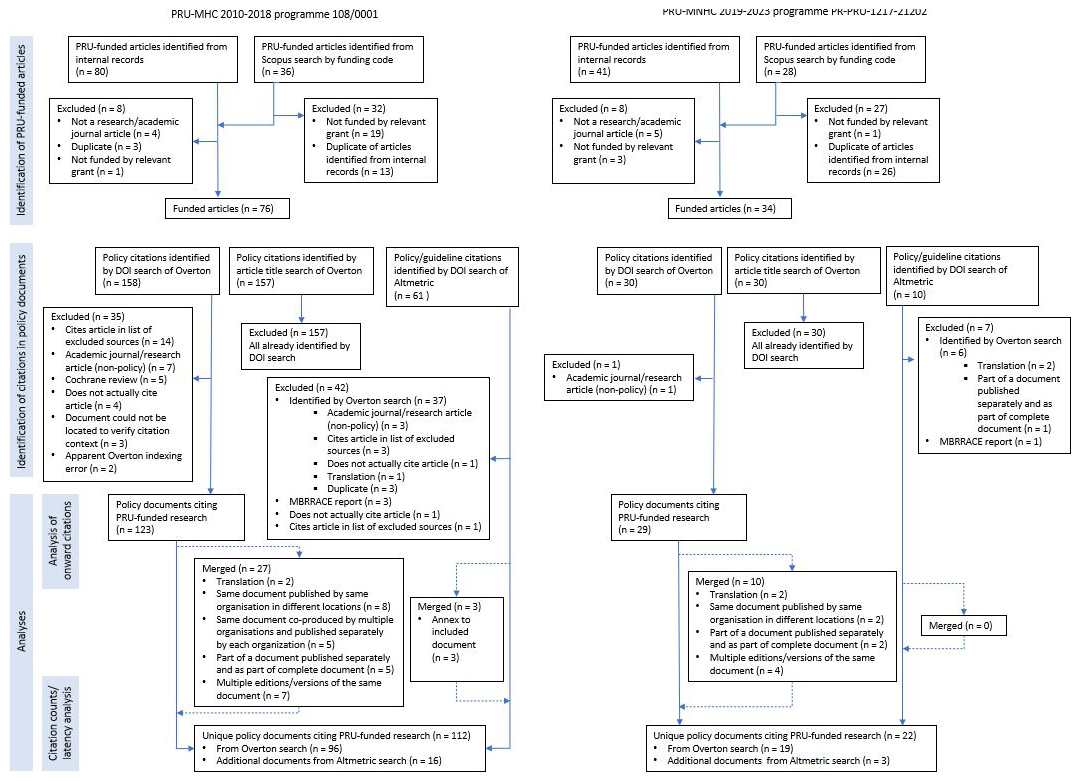
Study flow diagram.

Figure 2 shows the flow of articles and policy documents through the study.

### Identification of funded outputs

We identified 76 published articles funded by the 2010–2018 programme and 34 funded by the 2019–2023 programme as of 2 September 2024. Most of the articles were identified via internally held records. Using Scopus to search for the relevant grant codes returned only 17 of 74 articles funded by the earlier programme; four of these were not included in internal records. Searching Scopus also returned 19 articles that were funded by an unrelated grant from a different funder with the same code. Searching Scopus by grant code was somewhat more sensitive for finding articles funded by the later programme because of improved reporting of detailed grant information in the published articles. The search returned 27 of the 34 articles funded by the later programme, although only one of these was not already identified through internally-held records.

### Identification of citing policy documents and clinical guidelines

Searching Overton by the DOIs of the 110 PRU-funded articles returned an initial total of 188 policy documents (158 relating to articles funded by the 2010–2018 PRU programme, and 30 relating to articles funded by the 2019–2024 PRU programme), as of 17 September 2024. There was no additional value to searching Overton by the article titles – this did not identify any policy documents not already identified by searching by DOI, and failed to identify one document that was returned by the DOI search.

After all exclusions, the Overton search results were narrowed down to 115 unique policy documents citing PRU-funded articles (96 of which cited articles funded by the 2010–2018 programme and 19 of which cited articles funded by the 2019–2013 programme). The most common reason for excluding documents (n=14) was that examination of the citation context revealed that the PRU-funded article was cited in a list or appendix of excluded evidence; reasons for these exclusions included the article reporting on a study design, population, or outcome that differed from those of interest for the policy document.

Searching Altmetric for the DOIs of the 110 PRU-funded articles returned 71 policy documents and clinical guidelines (61 relating to articles funded by the 2010–2018 programme, and 10 relating to articles funded by the 2019–2024 programme), as of 2 December 2025. The majority of these citations (43/71) were also identified by the Overton search; searching Altmetric added an additional 19 unique citations to the total number identified (an increase of approximately 18%). Notable types of citing sources/types of citing documents among the citations identified by Altmetric that were not identified by Overton included clinical guidelines and consensus statements published in academic journals (n = 3) and several documents published by the World Health Organization (WHO) (n = 4).

### Characteristics of citations in policy documents of PRU-funded articles

**Figure 3.**
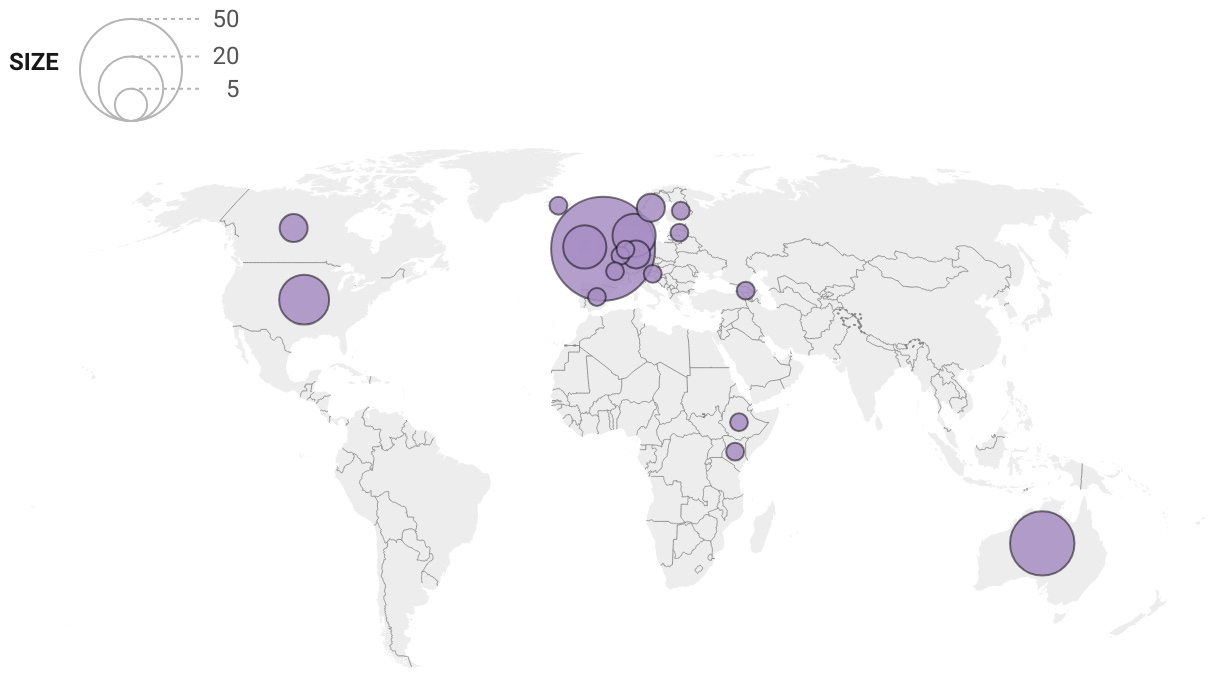
Geographic distribution of policy organisations that cited PRU-funded articles. Figure 3 footnote: Two citations by multi-national think tanks are visualised at both/all three of the relevant countries (Kenya/Denmark and USA/Sweden/Germany). An EU publication is localised to Brussels. Citations in documents published by IGOs are not visualised (n = 19)

Table 1 summarises the characteristics of citations in policy documents of PRU-funded articles. Citations were heavily skewed, with fewer than half of the articles being cited in policy documents, but a minority of articles being cited up to 25 times. Most citations were in documents authored by organisations based in the UK (52 of 134 citations) and other high-income countries as defined by the World Bank ( Figure 3). International IGOs accounted for around 15% of citations (20/134).

**Table 1.** Characteristics of citations in policy documents of PRU-funded articles.

	PRU grant ID		
	PRU-MHC 2010–2018 programme 108/0001	PRU-MNHC 2019–2023 programme PR-PRU-1217- 21202	Total
**Number of funded journal articles published**	76	34	**110**
**Number (%) published open access**	65 (86%)	34 (100%)	
Total number of unique * policy/clinical **guideline citations**	112	22	**134**
**Citations per article (median, range)**	0 (0–25)	0 (0–13)	**0 (0–25)**
**Number (%) of articles with ≥1 citation**	34 (45%)	9 (26%)	**43 (39%)**
**Type of citing organisation**			
Government	62	14	**76**
Think tank	26	1	**27**
Intergovernmental organisation	14	6	**20**
Clinical organisation	3	1	**4**
Other **	7	0	**7**
**Type of citing document**			
Policy publication	81	17	**98**
Clinical guidance	25	4	**29**
Working paper	6	0	**6**
Blog post	0	1	**1**
**Country of citing organisation**			
United Kingdom	43	9	**52**
Australia	15	4	**19**
North America	13	1	**14**
Ireland	8	0	**8**
Other Europe	15	2	**17**
Africa	0	1	**1**
EU	1	0	**1**
Georgia	1	0	**1**
Multiple countries	2 ***	0	**2**
International organisation	14	5	**19**
**Latency from article publication to citation in** **policy document (weeks) (median, range)**	205 (16–575)	47 (0.4–159)	**183** **(0.4–575)**
**Total number of onward policy citations** **(including by same organisation)**	622	49	**671**
**Total number of onward policy citations by** **different policy organisations**	378	96	**474**

*The following were counted as a single citation: multiple translations of the same document; the same document posted by the same organisation in multiple locations; the same document co-produced by multiple organisations and published separately by each organisation; a chapter of a document published alone and subsequently in a compendium; multiple editions of the same publication. For the purposes of onward citation counting, citations to all instances of each document were counted. **Includes independent research organisations, arm’s-length bodies ***Kenya/Denmark and USA/Sweden/Germany

Most citing documents (98/134) fell into the broad categorisation of ‘policy publication’, which includes documents such as reports by policy-relevant organisations, briefings to government committees, evidence syntheses conducted to inform policy, position statements, and governmental responses. Just over a fifth of citations (29/134) were in documents classified as clinical guidelines.

Policy documents that cited PRU-funded articles were themselves cited a total of 671 times in later policy documents (onward policy citations, Table 1).

### Policy citation timing and latency

**Figure 4.**
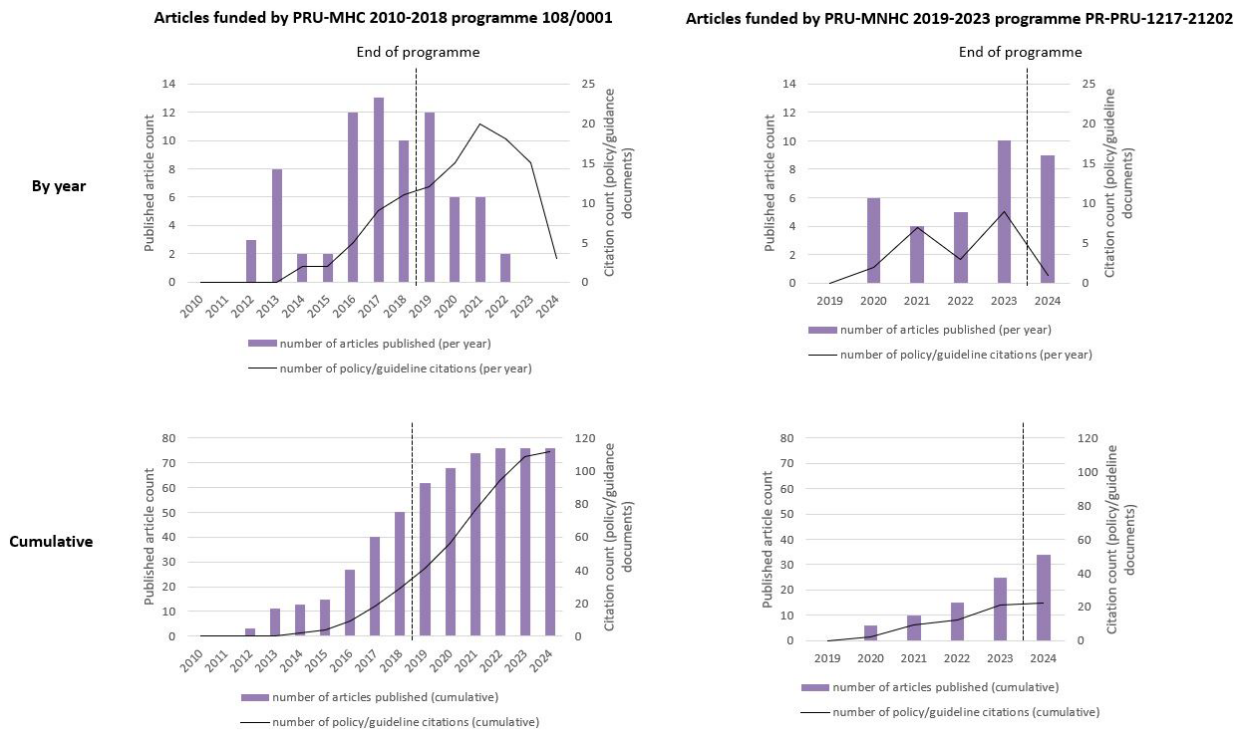
PRU-funded articles published and unique citations of these articles in policy documents over time.

Figure 4 shows the per-year and cumulative number of citations in policy documents to articles funded by each of the PRU programmes, over the lifetime of the programme and after the programme funding ended. This is plotted along with the number of journal articles published that were funded by the programme. The highest number of articles funded by the PRU-MHC 2010–2018 programme 108/0001 published in a single year occurred in the penultimate year of the programme (13 articles in 2017), and published outputs continued at a declining frequency in the four years after the programme ended. The largest number of policy citations in a single year came in the third year after the programme ended (20 citations in 2021). The highest number of articles funded by the PRU-MNHC 2019–2023 programme published in a single year occurred in the final year of the programme (10 articles published in 2023), and output remained high after the programme ended (9 articles published in 2024).

As of late 2024, the sixth year after the start of the PRU-MNHC 2019–2023 programme, 34 funded articles had been published and these had received 19 citations in policy documents overall. At the same point in the life of the earlier programme, six years after commencement of funding, 14 funded articles had been published that had been cited 4 times in policy documents.

**Figure 5.**
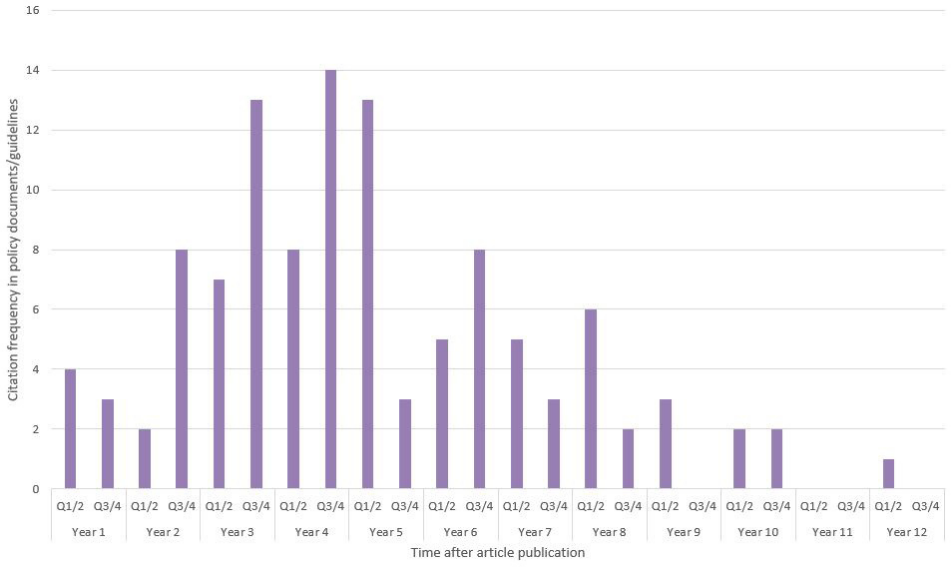
Latency to policy citation after publication of the cited article, for articles funded by the PRU-MHC 2010-2018 programme 108/0001.

The median latency from article publication to policy citation for articles funded by the first PRU programme was 205 weeks (range 16–575 weeks). The publication latency distribution is illustrated in Figure 5. As of September 2024, the median latency from article publication to policy citation for articles funded by the second PRU programme was 47 weeks (range 0.4–159 weeks), although this lower latency is influenced by the fact that most of the output for this programme was published in the past two years so no longer-term citations are yet possible.

### Citations in academic journal articles

**Figure 6.**
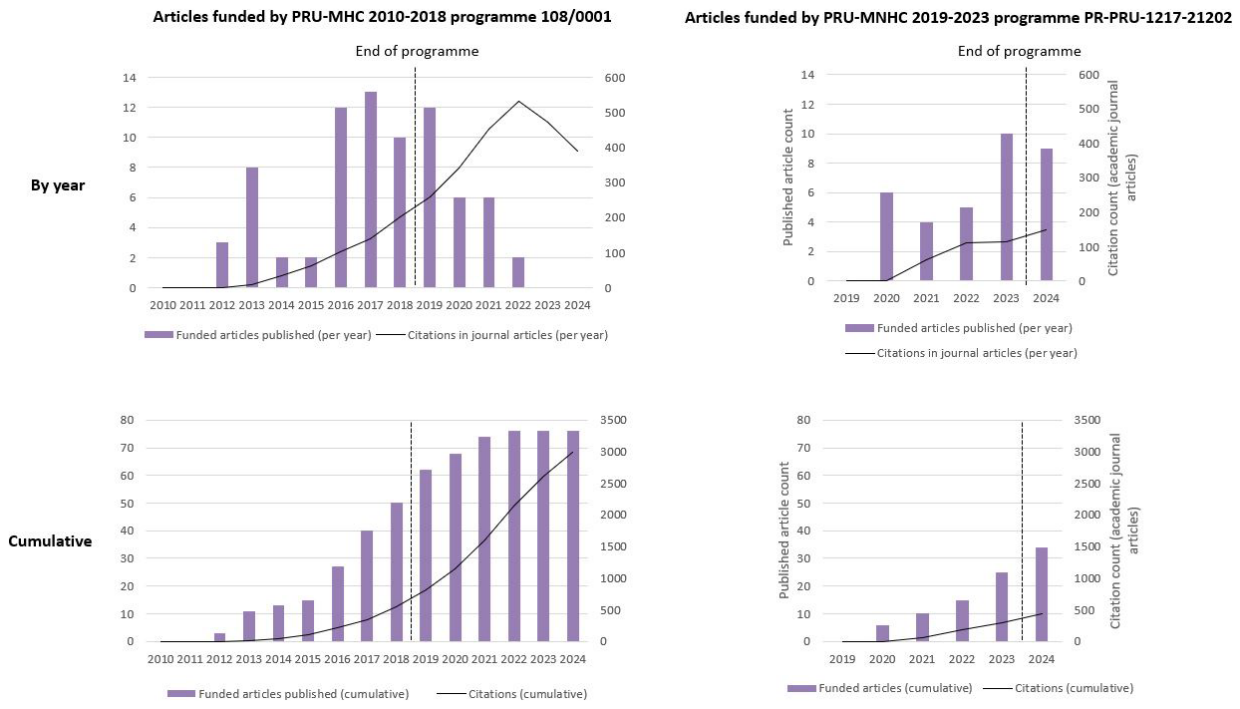
PRU-funded articles published and citations of these articles in academic journal articles over time.

In addition to exploring citations of PRU-funded research articles in policy documents, we quantified citations in academic journal articles ( Table 2). The 109 PRU-funded articles published to date that are indexed in Scopus have been cited a total of 3,435 times in journal articles (as of 7 November 2024); 103 of the 110 (94%) have been cited at least once, and the median number of citations to date per article is 17.5. Figure 6 shows the per-year and cumulative number of academic journal article citations to articles funded by each of the PRU programmes, over the lifetime of the programme and after the programme funding ended.

**Table 2.** Citations in academic journal articles.

	Articles funded by PRU-MHC 2010–2018 programme 108/0001 (n = 76)	Articles funded by PRU-MNHC 2019–2023 programme PR-PRU-1217-21202 (n = 33 *)	Total (n = 109)
**Total number of citations** ** in academic journal** ** articles**	2995	440	**3435**
**Citations per article** ** (median, range)**	23.5 (0–436)	4 (0–189)	**17.5 (0–436)**
**Number of articles with ≥1** ** citation**	74	29	**103**

*One PR-PRU-1217-21202-funded article (published in 2022) is not indexed in Scopus.

### Case studies

***Case study 1***

Article: Father involvement in early child-rearing and behavioural outcomes in their pre-adolescent children: evidence from the ALSPAC UK birth cohort, by Opondo *et al.* (2016). DOI: 10.1136/bmjopen-2016-012034 (funded by PRU-MHC 2010–2018 programme 108/0001)

This article was published in *BMJ Open* under a Creative Commons Attribution (CC BY 4.0) license (open access).

Characteristics of policy citations of this article are summarised in Table 3. Citing policy documents included reports, a literature review, a discussion paper, and a written evidence submission to a parliamentary committee. The article was cited twice in one document and once in the other seven. Citations were mainly in the context of providing background information on the nature and scale of the issue being discussed (e.g., ‘A study published earlier this year found that the children of active fathers were up to 28 per cent less likely to suffer behavioural problems in their pre-teen years compared to children without a father figure at home’. ^22^). One document included a more detailed summary of the methods, results and interpretation of the research ^23^, and another cited the study as motivation for an intervention (‘Another goal of the intervention is to improve father’s involvement in child rearing’ ^24^).

**Table 3.** Case study 1: characteristics of policy citations.

	n
**Unique citations in policy documents**	8
**Country of citing organisation**	
UK	7
Germany	1
**Type of citing organisation**	
Government	3
Think tank	5
**Type of citing document**	
Policy publication	7
Working paper	1
**Citations in academic journal articles**	64

The article was featured in a blog post published by *BMJ Open* on the most-read articles in November 2016 ^25^ and again in a post ‘highlights from 2016 in review’ as the third-most disseminated article of the year ^26^. A press release was published on 23 November 2016 by the University of Bristol, which provides core support for ALSPAC (the Avon Longitudinal Study of Parents and Children) ^27^.

The article was discussed in The Guardian (UK) on 28 November 2016 ^28^ and the BBC News website on 23 November 2016 ^29^, and cited in a piece in Good Housekeeping (UK) on 24 November 2016 ^30^. These media mentions predated all of the policy citations. The article was also cited in a Spanish-language article on The Conversation on 18 February, 2021 ^31^.

The article was mentioned in 127 X posts from 113 users.

Policy documents citing this article were themselves cited 41 times (19 times by organisations other than the original citing organisation). The most cited was a research report by the UK Government Equalities Office, ‘ *What motivates employers to improve their Shared Parental Leave and pay offers?’*

***Case study 2***

Article: Characteristics and outcomes of neonatal SARS-CoV-2 infection in the UK: a prospective national cohort study using active surveillance (funded by PRU-MNHC 2019–2023 programme PR-PRU-1217-21202).

The article was published in *The Lancet Child & Adolescent Health* under a Creative Commons Attribution (CC BY 4.0) license (open access).

Characteristics of policy citations of this article are summarised in Table 4. Citing policy documents included evidence summaries, reports, clinical guidance, and a manual and a field guide focused on vaccination. The article was cited once in eight of the 13 citing policy documents, twice in three, three times in one, and five times in one. The context of the citations included:

- Background on disease burden (e.g., ‘A prospective cohort study of 66 neonates infected with COVID-19 in the United Kingdom found that 42% were hospitalized with severe disease and 33% required respiratory support. However, only one death was reported, and it was not related to COVID-19’ ^32^.)
- Details of:
  ◦ incidence (e.g., ‘A population-based cohort study from the United Kingdom estimated the neonatal incidence of SARS-CoV-2 infection to be 5-6 [95% confidence interval 4·3–7·1] per 10,000 live births’ ^33^),
  ◦ risk factors (‘As seen with non-pregnant women, a high proportion of pregnant women have asymptomatic SARSCoV-2 infection and severe disease is associated with recognized medical (e.g., high bodymass index (BMI), diabetes, pre-existing pulmonary or cardiac conditions’ ^34^),
  ◦ presentation (‘Reported symptoms include rhinorrhoea, cough, lethargy, vomiting, diarrhoea, apnea, fever, tachycardia, tachypnea, leucocytosis, thrombocytopenia, hypoxemia, hypotension, raised C-reactive protein, elevated lactate and radiographic findings of ground-glass opacities’ ^33^),
  ◦ treatment (e.g., ‘Several case report studies described treating neonates, including premature infants, with remdesivir and observed clinical improvement and no adverse effects’ ^33^),
  ◦ outcomes (e.g., ‘Short-term outcomes were good, with 88 per cent of neonates discharged home, seven still in hospital and there was one death from an unrelated cause’ ^34^).
- A summary of key findings under a single citation including incidence, mode of infection transmission, outcomes, and evidence for disparities between ethnic groups (‘the incidence of infection was higher in babies from Black, Asian, mixed or other ethnic groups compared with babies from White ethnic groups: White: 4.6 babies per 10,000 livebirths; Asian: 15.2 per 10,000 livebirths; Black: 18 per 10,000 livebirths; Mixed or other ethnic groups: 5.6 per 10,000 livebirths.’)
- The article being mentioned as a resource that may be of interest to readers (e.g., under the heading ‘Peer reviewed journals featured’ in an evidence digest: ‘A UK cohort study on characteristics and outcomes of neonatal COVID-19 infection here [hyperlink]’ ^35^)
- Support for specific clinical practice recommendations (‘Potential aerial transmission should be minimized by wearing a mask and practicing hand hygiene’ ^36^; ‘infection with neonatal admission following birth to a mother with perinatal SARS-CoV-2 infection was unlikely, and possible vertical transmission rare, supporting international guidance to avoid separation of mother and baby’ ^37^)

**Table 4.** Case study 2: characteristics of policy citations.

	n
**Unique citations in policy documents**	13
**Country of citing organisation**	
UK	3
Australia	3
Iceland	1
Ethiopia	1
IGO	5
**Type of citing organisation**	
Government	6
IGO	6
Clinical organisation	1
**Type of citing document**	
Policy publication	11
Clinical guideline	2
**Citations in academic journal** ** articles**	189

A Eurek!Alert press release on the study was published on 9 November 2020 ^38^. The PRU-funded article was cited in the French-language Wikipedia ‘ *Maladie à coronavirus 2019*’ page on 8 April 2021 ^39^.

The article was discussed in the Daily Mail (UK) in an article published on 9 November 2020 ^40^. It was also cited in an article on The Conversation published on 1 March 2022 by researchers based at Swansea University in Wales, UK ^41^, and another published 26 May 2022 by Australia-based researchers ^42^. The story was also mentioned by a range of local/regional UK and US news outlets.

The article was mentioned in 426 X posts from 363 users.

Policy documents citing this article were themselves cited 41 times (13 times by organisations other than the original citing organisation). The most cited was a WHO scientific brief, ‘ *COVID-19 disease in children and adolescents*’. This brief was translated into French and Spanish, although all onward citations were to the English-language version. All other policy documents citing this PRU-funded article were in English, with the exception of a clinical guideline in Icelandic.

***Case study 3***

Article: Stillbirths: economic and psychosocial consequences, by Heazell *et al.* (2016). DOI: 10.1016/s0140-6736(15)00836-3 (this article was part-funded by PRU-MHC 2010-2018 programme 108/0001 and includes data extracted from an earlier report of a questionnaire study funded by the same PRU programme ^43^).

The article, which was part-funded by the PRU, was published in the Lancet as one of a series of five research papers and four comment articles titled ‘Stillbirths 2016: ending preventable stillbirths’. The series was produced in response to the Global Strategy for Women’s, Children’s and Adolescent’s Health, which was launched at the 2015 UN General Assembly and identified stillbirth as a key issue to tackle as part of the post-2015 sustainable development agenda ^44^.

Characteristics of policy citations of this article are summarised in Table 5. Citing policy documents included reports, economic analyses, submissions to parliamentary committees, clinical guidance/consensus statements/a handbook, and an information pack for parliamentary debate participants. The article was cited once in 13 documents, twice in eight, three times in one, eight times in one, and sixteen times in one (and the number of times cited in the final document, a clinical guideline in an academic journal, was unknown: the guideline’s reference list was available to confirm the citation but the full text was unavailable). The context of citations included:

- background (e.g., ‘Every stillbirth and death of a newborn baby is a tragedy and has a devastating impact on bereaved parents and families, who have higher rates of depression and anxiety and often do not receive the care and support they need’) ^45^
- inclusion of a reprinted or modified figure illustrating the impact of stillbirth ^46, 47^
- data extracted from the article used in multiple analyses in an economic audit ^48^
- identification of an avoidable risk factor, for which specific clinical recommendations were then provided (‘The Bundle addresses commonly reported care factors that can be moderated including…for women with decreased fetal movements (DFM), maternal sleep position (a recently reported avoidable risk factor for stillbirth)’) ^49^
- quotations from study participants included in an evidence submission to a parliamentary committee ^50^
- specific evidence underpinning cost-benefit analyses (e.g., ‘Lastly, we value stillbirths as the avoided healthcare provider and patient costs associated with the stillbirth, including the cost of increased complications for the birth and an additional pregnancy. A study by Heazell *et al.* (2016) suggests that care costs for stillbirths were 10–70% greater than with a live birth and we use the midpoint of 40% to estimate the increase in costs associated with a still birth’ ^51^; ‘Direct healthcare costs are assumed to be GH¢ 2800, on the basis that stillbirths cost 40% more than live births (Heazell *et al.* 2016) and the average cost of a hospital live birth is GH¢ 2000.’) ^52^

**Table 5.** Case study 3: characteristics of policy citations.

	n
**Unique citations in policy documents**	25
**Country of citing organisation**	
UK	4
Australia	8
Ireland	2
USA	3
Denmark	1
Kenya/Denmark	1
IGO	5
**Type of citing organisation**	
Government	14
Think tank	4
IGO	5
Clinical organisation	1
Other	1
**Type of citing document**	
Policy publication	23
Working paper	1
Clinical guideline	1
**Citations in academic journal articles**	436

A EurekAlert! press release on the Lancet series was published on 18 January 2016. The PRU-funded article was cited in the English-language Wikipedia ‘ *Stillbirth*’ page on 30 August 2016. This predated all but one of the 25 policy citations.

The article was cited in an article on The Conversation published 25 October 2017, written by a group of Australia-based academics ^53^. This predated all of the policy citations by Australian organisations.

The article was mentioned in 60 X posts from 68 users; all but 3 of the posts were from 2016 (37 within the first week after publication).

Policy documents citing this article were themselves cited 58 times (38 times by organisations other than the original citing organisation). A UNICEF report, ‘ *A Neglected Tragedy: The global burden of stillbirths. Report of the UN Inter-agency Group for Child Mortality Estimation, 2020*’ was the most cited.

## Discussion

### Use of PRU-funded research in policy and clinical guidelines

We found that almost half of articles published under the 2010–2018 PRU-MHC programme, and a quarter of those published under the 2019–2023 PRU-MNHC programme, were cited in at least one policy-relevant document or clinical guideline. The majority (around 85%) of the included unique citations were identified via Overton, with Altmetric searches yielding the remainder. This policy uptake frequency compares favourably with the findings of a previous study using Overton that estimated that 6.0% of all publications, and 8.6% of those from the UK, that were funded by the European Commission’s 7 ^th^ Framework between 2007 and 2013 received at least one citation in a policy-relevant document ^15^. The same study estimated that approximately 13% of all Scopus-indexed healthcare research articles published in 2008 had been cited in policy at least once by the time the study was conducted in 2021, falling to around 6% for articles published in 2016 (with this lower proportion reflecting citation lag).

How the citing documents used the PRU research varied, from provision of general background information and more detailed summaries of findings to support for specific clinical recommendations and use of specific evidence as the basis for the methodology used in economic analyses.

### Differences between the first and second PRU programmes

Compared with the first PRU programme (2010–2018) over the equivalent time period (i.e., during the six years after funding commenced), the second PRU programme (2019–2024) generated more publications (34 vs 15), more policy citations (22 vs 4), and more citations in academic journal articles (440 vs 108). The difference in policy citations may in part reflect an under-ascertainment of older policy sources owing to the limitations of Overton’s indexing ^54^. However, it is also likely that policy citations to research produced under the second PRU will continue to accumulate. For research published under the first PRU, the median delay from article publication to citation in policy was around four years; most of the articles published under the second PRU were published within the two years preceding this analysis.

The high number of policy citations to date for articles funded by the second PRU in large part represents citations of research relevant to SARS-CoV-2/Covid (16 of the 22 policy citations identified). Under the atypical conditions of the pandemic, PRU-funded research likely had more impact, faster, than might have been expected under normal circumstances, and citation patterns during this period are unlikely to be generalisable. However, this finding highlights the fact that enhanced use of research evidence in policy is possible when research and policy agendas align and evidence-based healthcare policy is a national priority. There is potential to build on lessons learned, and practices and networks established, during the pandemic and apply these to other topics within the healthcare research–policy–clinical practice nexus ^55^.

The apparent increased use of PRU evidence in policy under the second programme of funding may also reflect strategic efforts by the PRU to build relationships with policymakers in relevant areas. These efforts have included increasing policymaker involvement throughout the research cycle, from initial choice of research questions through to dissemination and evaluation; and embedding researchers in policy groups and clinical organisations where they can have input into policy priority-setting and disseminate findings. Increased evidence use in policy could also reflect a greater awareness of policy research by policymakers as a consequence of development of the national policy research landscape - the numbers of NIHR Policy Research Units grew during this time to 17, so work of such Units might have had greater visibility in the policy arena. We also considered whether increased accessibility of research to policymakers as a consequence of mandatory open access publishing might be operating as a factor (it has been required since 2014 that reports of main findings of NIHR-funded research be published under an open access licence, and this mandate was extended in 2022 to cover all findings ^56^). However, the proportion of articles funded by the first PRU programme that were cited in policy did not differ between those published under a Creative Commons open access license and those not. Every article funded by the second PRU programme was published under a Creative Commons open access licence.

We suggest that the higher rate of article publication emerging from the second PRU programme might reflect increased efficiency of an established PRU facilitating increased productivity, pointing to development of capacity for policy-relevant research as an impact of the NIHR PRU funding model ^57^. We are confident that our lists of articles funded by each PRU programme were complete or nearly complete, owing to stringent in-house record-keeping; however, fewer than a quarter of articles funded by the first PRU programme were detectable via a Scopus search for the relevant grant code. As a general lesson learned on good practice, including consistent and detailed funding declarations in published articles facilitates retrospective identification of funded outputs for bibliometrics approaches.

### Areas for improvement

Although our findings on policy citation frequency are encouraging when considered in the context of healthcare research as a whole, research conducted by the PRU is specifically designed to be relevant to national policy. We should therefore reflect on why over half of PRU-funded published articles have not yet received any detectable policy citations. Our findings cannot explain this, but possible factors to consider include a mismatch between the research and policy agendas, changing policy priorities, speed of research delivery relative to policy timelines, a lack of accessibility or visibility of published research, or underascertainment of citations owing to a lack of publicly available or indexed policy documents ^6^. Although we cannot demonstrate a causal effect, it is interesting to note that in our second and third case studies of highly-cited articles, coverage in non-academic outlets including news media and Wikipedia preceded policy citations; this coverage may have enhanced visibility and/or accessibility of the research to policymakers. The articles that these case studies focus on were also the subject of features in their publishing journals, which could reflect an existing high level of interest in the topic and/or could have served as a route to enhancing visibility. Neither Overton nor Altmetric tracks mentions of research by charities/third-sector organisations as a distinct category, but this would be worth exploring as a route that the PRU might be able to make better use of to disseminate and amplify research outputs and make them more visible and accessible to policymakers.

It is worth noting that around 7.5% of policy citations identified in our initial searches (14/188) related to mentions of a PRU-funded article as an excluded source. For eight of these articles, no eligible citations in policy sources were identified. These might suggest ‘missed opportunities’: the topic of the research was potentially relevant to a policy document, but another aspect of the study such as population or design meant it ultimately could not be used. In some cases, this may reflect requirements for inclusion that would have been unfeasible or impractical for the PRU research study (e.g., comparative design for certain questions; non-UK population of interest). However, we hope that strengthening relationships between researchers and funders will improve understanding of evidence needs at the point of study inception and maximise the utility of research outputs.

The time lag from research publication to citation in policy documents and guidelines, observed here and by others ^15^, represents another aspect of policy research with potential for improvement. Given that PRU-funded research is, for the most part, designed to meet current or anticipated imminent policy evidence needs, the observed median four-year delay to citation in policy documents is perhaps surprising. There is a clear ethical argument for rapid, efficient use of healthcare research to maximise benefit to patients and minimise delay and research waste. Beyond this, citation lag also has practical implications for researchers’ individual careers and sustainability of the research workforce. Researchers are increasingly expected to demonstrate the utility, benefit and impact of their research in order to secure funding renewals and new or follow-on grants ^58, 59^. The delay we observed between research publication and use in policy documents highlights one reason why this can be challenging, especially in a funding landscape dominated by short-term grants. Demonstrable impacts on healthcare system improvements and patient benefit, where they occur, are likely to follow even later. In addition to improving relevance of evidence to policy, we hope that strategic efforts to build relationships between researchers and policymakers will speed up the pathway from evidence to policy to impact. A more positive framing of the observed citation lag would be that the usefulness to policymakers and the potential impact of research continues long after the funding cycle has ended.

### Citation tracking as a methodology for evaluating the use of research in policy and clinical guidelines

Quantitative analysis of citations in policy documents provided a sense of the reach and relevance to policy of research conducted by the PRU, and also revealed the issue of citation lag. Comparing the outputs and citations of the two PRU funding programmes also suggested capacity building or improved efficiency as possible outcomes of sustained PRU activity. As such, the citation tracking and analyses undertaken here delivered considerably more insight into evidence uptake and other possible outcomes of PRU activity than simple citation counting alone would provide. However, the approach was constrained by some of the limitations inherent in citation metrics. We did not attempt to systematically categorise or quantify how and to what extent each cited article contributed to or influenced the message of the citing policy document, or the influence of the policy documents on legislation or practice. The bibliometric approach to tracking research use is also dependent on policymakers citing research evidence when it informs policy, which does not always happen. Unlike scholarly publishing, which has established standards for referencing, citation practice in policy documents is much more varied. Agreement between researchers and policymakers on a standardised approach to recognising and citing evidence could be mutually beneficial and facilitate bibliometric tracking of the evidence–policy relationship.

More in-depth exploration of the citation context in our case studies provided somewhat more insight into areas where use of PRU research might ultimately have downstream effects on clinical practice or meaningful changes to policy. For example, in case study 2, one document cited the PRU article in support of not separating a mother with perinatal SARS-CoV-2 infection from her newborn – a recommendation which, if followed, has clear implications for clinical practice and patient wellbeing. In case study 3, a report from the United Nations Population Fund cited the PRU research in support of recommendations including provision of paid leave from employment for parents who experience a stillbirth ^46^. Another document discussed disparities in outcomes between babies of different ethnicities that were demonstrated by the research; such findings, highlighted by a politically influential entity (the UK’s Parliamentary Office of Science and Technology), might be expected to influence further policy discussions. Such areas of evidence use could be explored further to determine whether they led to meaningful impact. However, without further investigation these examples fall short of providing concrete evidence of impact of PRU research, defined by the NIHR as ‘the demonstrable contribution that research makes to society and the economy, benefiting individuals, organisations, and nations’ ^60^, which is the ultimate aim of policy-focused healthcare research. Tracking a direct line from evidence to policy/recommendations, and from there to practice and patient benefit, would likely need to involve discussion with policymakers to confirm whether and how much the evidence contributed. Such investigation would best be done contemporaneously, because memories are likely to fade over time when evidence use is not documented, and individual policymakers move on to new positions.

It should also be noted that while our approach investigated citations of published articles only, the outputs and activities of the PRU-MNHC with potential to influence policy are diverse and include reports, information briefings, infographics, talks, and participation in roundtables and other meetings. It is more difficult to track use of such materials and activities: insights founded on research may be used to inform policy but not cited, or may be referenced in a form that is not indexable. Thus, bibliometric monitoring and citation analysis using tools such as Altmetric and Overton can offer only part of the picture of how research is used in policy: sustained relationships with policymakers and involvement of researchers in policy groups are needed to allow for planning, dialogue and feedback.

A notable example of impact of PRU-funded research that our citation tracking approach failed to detect is the case of 6-week postnatal checks in England. A PRU project conducted in response to a request from NHS England found disparities in the provision of the NICE (National Institute for Health and Care Excellence)-recommended maternal postpartum 6-week check. This finding was based on analysis of women’s primary health care records from 2015–2019. The evidence led to a 2019 meeting between NHS England and the British Medical Association and an agreement that provision of 6-week checks would be an essential service included in the new GP (general practitioner/family doctor) contract, with an additional GBP 12 million of funding to support provision. The requirement was enacted in 2020. However, our search did not identify any citations related to this impact in policy documents or clinical guidelines. The research findings were communicated to NHS England in an unpublished report prior to journal publication; the requirement for 6-week checks was enacted in 2020, before the journal article describing the underlying evidence ^61^ was submitted for publication (in February 2021) and first made available online (in September 2021). In this example the evidence–policy–practice relationship worked very well, but could not be evidenced by our citation tracking approach: the impact predated article publication and as of March 2025, the journal article has not received any citations in policy documents detected by Overton or Altmetric. We plan to start depositing unpublished outputs such as reports and policy briefs in an archive where they can be assigned a digital object identifier (DOI); although this will not resolve the issue entirely, it should make grey literature easier to cite, and citations easier to index.

### Limitations of Overton and Altmetric as sources of policy citations

This review was limited by the limitations of Overton, including omission of any citing sources that exist behind a paywall or login. It also identified only policy documents that have been published online, and by one of Overton’s extensive but non-exhaustive list of sources. Detection of citations in the early years of PRU funding, in particular, may be incomplete: Overton states that, as a general rule, ‘coverage is generally good from 2015 onwards and sparse before 2009’ ^54^. Policy documents in languages other than English were also less likely to be identified ^12^. The apparent dramatic drop-off in 2024 in the year-on-year policy citation count (beyond what might be expected of a post-Covid normalisation) also suggests that there may be an indexing lag, so recent citations may not have been detected. Each of these limitations could result in an underestimated citation count.

Altmetric citation tracking is very likely limited by the same factors as Overton, although available information about Altmetric methodology is more limited. Both Altmetric and Overton purport to track mentions of research articles in similar types of document. We found that Overton detected more citations; the results from Overton and Altmetric overlapped, but each tool detected citations that the other did not. Thus, using both tools in tandem is worthwhile to maximise search sensitivity. Notably, Overton aims not to index academic journal articles; in keeping with this, we found that Altmetric identified several citations in clinical guidelines that were published in academic journals, which Overton did not index.

A relatively high proportion of results returned by our initial searches of Overton and Altmetric were excluded from our count of unique citations – around a third of the results from each tool, not including duplicate results that were excluded because they were returned by both tools. This was because they either did not meet our inclusion criteria (e.g., the article was cited in the policy document in a list of excluded evidence, or was cited in an academic journal article rather than a policy document), or because they substantially overlapped with other returned documents (e.g., identical documents posted in different locations online, or multiple editions of the same document). Therefore, checking citation context is recommended to avoid overestimating the relevance of research to policy, which increases the labour-intensiveness of this approach.

The data that we report on the types of citing document and organisation used the categorisation provided by Overton and Altmetric (or in the case of organisation type for Altmetric results, manual categorisation based on organisational websites). These categories are broad and lack clear definition. As such, these data provide a sense of the types of citing documents and organisations in very general terms, but do not support detailed analyses or conclusions. The analysis of latency from publication to citation was also reliant on the accuracy of the policy document publication dates as indexed by Overton or Altmetric. Additionally, we did not review individual onward citations in context because of the extensive resources that this would have required, so this count may be an overestimate.

### Other limitations of this study

When preparing our list of published PRU outputs, we did not discriminate between articles fully and partially funded by the PRU, or look at the relative contribution of PRU funding where there were multiple funders. Our analysis of citations in journal articles is limited by use of a single index (Scopus) to obtain citation counts, and citation context was not examined.

While the bibliometric approach provided insight into evidence use in policy as an outcome of PRU activities, this is not the only form of outcome that we would like to be able to track. PRU research frequently leads to leveraged funding for other studies. Such follow-on funding can itself be considered an outcome of PRU activities; furthermore, these studies may then go on to themselves have impact, which would not be easily traceable back to the original PRU research by bibliometric analysis.

## Conclusions

Overton and Altmetric are useful tools for evaluating the reach of healthcare research and its relevance and use for policy and clinical practice, although checking for overlapping and irrelevant results increases the resource demand. The approach also highlighted the delay between evidence publication and uptake in policy as an area with potential for improvement. However, retrospective quantitative citation analysis is of minimal use for describing impacts of research on clinical practice and patient wellbeing. In-depth examination of citation context provides insight into potential areas of policy influence, but still falls short of evidencing true impact and is resource-intensive. A more in-depth understanding of evidence use is likely to come from building strong relationships and ensuring frequent dialogue between researchers and key policymakers, and ensuring contemporaneous investigation and recording of the use of research and its impacts where these emerge. Citation analysis could be used as an adjunct approach to detect unanticipated uptake and broader reach and longevity of evidence in policy applications.

## Ethics and consent

No ethics approval or consent was required for this retrospective analysis of publicly available documents.

## Data Availability

Because Overton, Altmetric and Scopus are commercial databases, the underlying data to this research cannot be shared openly due to copyright licensing restrictions. The Methods section contains detailed information to allow replication of the study. Requests to access the data can be made by contacting the National Perinatal Epidemiology Unit data access committee via general@npeu.ox.ac.uk and will be considered in the context of the conditions of the database licences. The estimated response time for requests is 4 weeks. For more information about procedures and conditions for data access, please refer to the National Perinatal Epidemiology Unit Data Sharing Policy available at https://www.npeu.ox.ac.uk/assets/downloads/npeu/policies/Data_Sharing_Policy.pdf.

## Software availability statement

All statistical analyses were performed using Microsoft Excel, a commercial software. All analyses in this study can be replicated using freely available statistical software such as LibreOffice Calc.
