# Supplementary material for: Evaluating Overton and Altmetric as tools for tracking healthcare research use and impact on policy and practice: a descriptive study
Source: NIHR Open Res. 2025 Jul 16;5:59. [Version 1] doi: 10.3310/nihropenres.13999.1 (PMC12780511; doi:10.3310/nihropenres.13999.1)
Supplement: Peer review history

# Reviewer report: Reviewer response for version 1

**DOI:** 10.3310/nihropenres.15221.r38809

**Published:** 2026-01-07

**Authors:** Wenceslao Arroyo-Machado

**Affiliations:** 1 Ingenio CSIC-UPV (Ringgold ID: 117010), Valencia, Valencian Community, Spain

The authors have conducted an interesting and novel study in which, using the two main providers of policy document citations (Overton and Altmetric), they carry out a case study with the NIHR Policy Research Unit for Maternal and Neonatal Health and Care. The authors have made a commendable effort in tracking, verifying, and combining policy citations, conducting a descriptive study and then focusing on several cases to contextualise and better understand the phenomenon. This is a work that may be of great use to the community wishing to explore this type of attention, particularly given the level of detail provided by the authors, and it requires only minor adjustments.

 As the main recommendation, I would highlight the approach and structure of the work. The title and objectives of the study (evaluating both Overton and Altmetric) create a reasonable expectation of comparison between these two major data providers. The authors make a notable effort in locating and processing the policy citations, but such a comparison is never directly undertaken. Whilst it is true that the authors provide all the details throughout the manuscript (Figure 2 is very detailed in this regard) a section in the results that addresses this matter head-on is lacking.

 The authors could therefore make better use of what they already have to structure the content more effectively, separating the evaluation from the case study. To this end, a comparative section could include elements such as:

1. the degree of overlap in policy document citations between platforms,
2. a quantified comparison of errors classified by severity (serious aggregator errors such as assigning citations where none exist, or minor errors such as incorrectly classifying document types),
3. what types of documents each tool detects that the other does not,
4. or differences in temporal and geographical coverage.

This would allow readers to understand how complementary (or not) both tools are, and those interested in this type of study could better comprehend which tool may be more suitable for each case or how much they might miss depending on their choice, especially considering that researchers typically do not have simultaneous access to both tools. Indeed, this would align very well with what the authors already discuss, but would offer quantifiable results regarding the similarities and differences between both providers.

 Minor issues

- The title is too broad and ambiguous, suggesting that an entire discipline is being studied when it is actually a very specific case. It would be appropriate to be somewhat more specific.
- In the introduction, a specific coverage date is provided for Altmetric, but the same information is missing for Overton.
- The authors correctly frame their work within altmetrics in the keywords, but there is no mention of this in the manuscript. It is suggested that at least one or two lines be added in the introduction explaining that this falls within the field of altmetrics and what this entails, so that those approaching this work with an interest in this area can familiarise themselves with it. Here, the authors could cite the Altmetrics Manifesto by Priem et al. (2011)( https://digitalcommons.unl.edu/cgi/viewcontent.cgi?article=1187&context=scholcom)
  as a seminal resource, the review by Sugimoto et al. (2017) which consolidates the original work on altmetrics and serves as a key reference, and the work by Thelwall (2020) which explains the different metrics, their meanings, and common problems as a resource that clarifies their use in research evaluation.
- This topic (the use and comparison of Overton and Altmetric for policy citations) has few existing studies. The Maleki & Holmberg (2024) work may be of use to the authors for introducing or discussing their research.
- The first two subsections of the results, "Identification of funded outputs" and "Identification of citing policy documents and clinical guidelines", essentially quantify the data retrieval and processing procedure. It might be simpler to include this information directly in the methods section when describing the process, rather than repeating the same information twice, once without figures and once with them.
- Figure 3 has points that, due to their size, overlap and prevent the exact location from being seen, although it can be intuited. It is recommended that the authors use a more adjusted scale or, if this makes it difficult to appreciate, that they simply colour the countries instead.

Is the work clearly and accurately presented and does it cite the current literature?

Partly

If applicable, is the statistical analysis and its interpretation appropriate?

Yes

Are all the source data underlying the results available to ensure full reproducibility?

Yes

Is the study design appropriate and is the work technically sound?

Partly

Are the conclusions drawn adequately supported by the results?

Yes

Are sufficient details of methods and analysis provided to allow replication by others?

Yes

Reviewer Expertise:

Scientrometrics

I confirm that I have read this submission and believe that I have an appropriate level of expertise to confirm that it is of an acceptable scientific standard.

## References

[1] : Scholarly use of social media and altmetrics: A review of the literature. *Journal of the Association for Information Science and Technology* .2017;68(9) : 10.1002/asi.23833 2037-2062 10.1002/asi.23833

[2] : The Pros and Cons of the Use of Altmetrics in Research Assessment. *Scholarly Assessment Reports* .2020;2(1) : 10.29024/sar.10 10.29024/sar.10

[3] : Policy Citations tracked by Overton.io versus Altmetric.com: Case Study of Finnish Research Organizations in Social Sciences. *Informaatiotutkimus* .2024;43(3-4) : 10.23978/inf.145570 4-28 10.23978/inf.145570

# Reviewer report: Reviewer response for version 1

**DOI:** 10.3310/nihropenres.15221.r38690

**Published:** 2026-01-07

**Authors:** Ashraf Maleki

**Affiliations:** 1 University of Turku, Turku, Finland

This paper has quantitatively analyzed extended impact of 110 publications funded by two programs in PRU in two time frames of 2010-2018 and 2019-2023 for mainly policy citations indexed by Overton.io and Altmetric.com but also some other media outlets for three cases studies. The paper is generally well-designed and well-written, and is relatively informed about issues of the citations counts obtained from Overtion.io and altmetric.com for policy to research citations. The comments below will hopefully help to improve the research:

 1. There is a mismatch in dates of data covered in pages 7 and 8 in PDF version. Most places refer to second period as “2019-2023” which suddenly changes to “articles funded by the 2019-2024 program” in the paragraph that begins with “Searching Altmetric…” which has been performed December 2025.

 2. Paper only refers to frequencies in most places. It would be useful to refer to some statistics such as exclusions in percentage form. This can be important given that policy citations often tend to shrink due to negative or exclusionary citations and non-existing ones. Please mention how many (%) funded journal articles were ultimately influenced by excluded citations. I would also propose reporting the average percentage of citations getting excluded at article level, if many articles experience it.

 3. Author-“Compared with the first PRU programme (2010–2018) over the equivalent time period (i.e., during the six years after funding commenced), the second PRU programme (2019–2024) generated more publications (34 vs 15), more policy citations (22 vs 4), and more citations in academic journal articles (440 vs 108). ” Comment- This statistics is not clearly communicated in the findings. Figure 5 only reports latency to policy citation for one program, 2010-2018. The figure for the following program (e.t. 2019-2023) is missing so it is difficult to follow the discussion in relation to findings reported. Likewise about citation from journal articles. The lack of clear related findings also influence understanding the later statement “We suggest that the higher rate of article publication emerging from the second PRU programme might reflect increased efficiency of an established PRU facilitating increased productivity, pointing to development of capacity for policy-relevant research as an impact of the NIHR PRU funding model”

 Provide clearer comparison between the two programs in terms of accumulation of citations from policy and other journal articles in the (six-year) chosen time period would improve supporting the discussion and these distinct statements.

 4. Finally, the references are rather remote and mostly non-peer reviewed work. There is an increasing body of research on policy citations to research and mostly still are only in preprint level, but some of them are closely connected and published in peer reviewed journals and conferences that will improve the discussion. Policy content analysis:

 A Maleki, M Lehtisaari, K Holmberg (2025). How Scientific Research Impacts Policy Cycle. The proceeding of the 20th International Conference on Scientometrics & Informetrics, 928-950. https://doi.org/10.51408/issi2025_055

 Comparison between Overton and Altmetric.com results:

 (Refer ref no. 1)

 Also, perhaps the preprint below due to its institutional focus:

 Torres Salinas, D., Arroyo Machado, W., Robinson García, N., & Romero Frías, E. (2025). From science to policy: A multilevel framework for measuring institutional policy influence through Overton. https://doi.org/10.5281/zenodo.15594572

 Overall, the paper looks well-written and I was impressed by the detailed break-down of policy citation types, unlike the discussion. The findings were not (clearly) showing the increase it claims across the two programs. This part requires improvement by making changes in the presentation of findings or modifying the discussion.

Is the work clearly and accurately presented and does it cite the current literature?

Partly

If applicable, is the statistical analysis and its interpretation appropriate?

No

Are all the source data underlying the results available to ensure full reproducibility?

Partly

Is the study design appropriate and is the work technically sound?

Yes

Are the conclusions drawn adequately supported by the results?

No

Are sufficient details of methods and analysis provided to allow replication by others?

Yes

Reviewer Expertise:

Altmetrics; Scientometrics

I confirm that I have read this submission and believe that I have an appropriate level of expertise to confirm that it is of an acceptable scientific standard, however I have significant reservations, as outlined above.

## References

[1] : Policy Citations tracked by Overton.io versus Altmetric.com: Case Study of Finnish Research Organizations in Social Sciences. *Informaatiotutkimus* .2024;43(3-4) : 10.23978/inf.145570 4-28 10.23978/inf.145570

# Reviewer report: Reviewer response for version 1

**DOI:** 10.3310/nihropenres.15221.r38687

**Published:** 2025-12-29

**Authors:** Steffen Lemke

**Affiliations:** 1 Kiel University, Kiel, Germany

The authors present a highly interesting investigation of the usefulness of Overton and Altmetric as instruments for gaining insights into the uptake of (health) research in policy documents. They perform a respective quantitative analysis for all publications associated with two past PRU Programmes. As part of this analysis, the authors conducted extensive manual full-text screening of respective policy documents to determine citation contexts. To round their analysis up, they present three more detailed case studies of highly cited publications from their sample. 

 I believe the article to be valuable for multiple reasons (and stakeholders): Firstly, the article's discussion highlights several highly relevant issues regarding the transfer of academic research into policy and practice, and along with it gives input on how to improve the situation. Secondly, users of Overton/Altmetric can take from this study a lot of helpful considerations regarding how to adequately present data about policy citations of academic publications, as well as related limitations to keep in mind.

 The authors' description of their data collection and processing is transparent and thorough. I in particular want to commend the detailed yet easily comprehensible presentation of their data workflow (depicted in Figure 2), which does a great job of informing about various peculiarities and/or limitations that analysts of policy citation data should consider when utilizing data sources like Overton or Altmetric for use cases similar to the one pursued in this article.

 For future work following up on this article, I want to make two suggestions: 

 1) The authors noted high numbers of "onward policy citations" (see for instance Table 1) in their analysis, which (understandably) could not be investigated in detail as part of this study. I think it will be interesting for future studies to focus more closely on this phenomenon, i.e. trace the flows of scholarly findings throughout the policy & practice landscape across multiple levels in greater detail. 

 2) It might be interesting to include a parametrization of publishing journals' reputation/impact into this kind of analysis, i.e. investigate its potential relationship with the characteristics of policy citations of respective articles.

Is the work clearly and accurately presented and does it cite the current literature?

Yes

If applicable, is the statistical analysis and its interpretation appropriate?

Not applicable

Are all the source data underlying the results available to ensure full reproducibility?

Partly

Is the study design appropriate and is the work technically sound?

Yes

Are the conclusions drawn adequately supported by the results?

Yes

Are sufficient details of methods and analysis provided to allow replication by others?

Yes

Reviewer Expertise:

Bibliometrics, Information Science, Scholarly Communication

I confirm that I have read this submission and believe that I have an appropriate level of expertise to confirm that it is of an acceptable scientific standard.
